# Design and Characterisation of pH-Responsive Photosensitiser-Loaded Nano-Transfersomes for Enhanced Photodynamic Therapy

**DOI:** 10.3390/pharmaceutics14010210

**Published:** 2022-01-16

**Authors:** Sooho Yeo, Il Yoon, Woo Kyoung Lee

**Affiliations:** Center for Nano Manufacturing and Department of Nanoscience and Engineering, Inje University, Gimhae 50834, Korea

**Keywords:** photodynamic therapy, photosensitisers, nano-transfersomes, pH-responsive drug delivery system, anti-cancer therapy

## Abstract

Photodynamic therapy (PDT) is a non-invasive and tumour-specific therapy. Photosensitizers (PSs) (essential ingredients in PDT) aggregate easily owing to their lipophilic properties. The aim of this study was to synthesise a PS (methyl pheophorbide a, MPa) and design a biocompatible lipid-based nanocarrier to improve its bioavailability and pharmacological effects. MPa-loaded nano-transfersomes were fabricated by sonication. The characteristics of synthesised PS and nano-transfersomes were assessed. The effects of PDT were evaluated by 1,3-diphenylisobenzofuran assay and by measuring photo-cytotoxicity against HeLa and A549 cell lines. The mean particle size and zeta potential for nano-transfersomes ranged from 95.84 to 267.53 nm and −19.53 to −45.08 mV, respectively. Nano-transfersomes exhibited sustained drug release for 48 h in a physiological environment (as against burst release in an acidic environment), which enables its use as a pH-responsive drug release system in PDT with enhanced photodynamic activity and reduced side effects. The formulations showed light cytotoxicity, but no dark toxicity, which meant that light irradiation resulted in anti-cancer effects. Additionally, formulations with the smallest size exhibited photodynamic activity to a larger extent than those with the highest loading capacity or free MPa. These results suggest that our MPa-loaded nano-transfersome system is a promising anti-cancer strategy for PDT.

## 1. Introduction

Cancer is one of the greatest health concerns worldwide [1,2]. Cancer is a devastating disease that results from uncontrollable growth of cells and their subsequent spread across the body [3,4,5]. Currently, various therapeutic strategies for cancer, such as surgery, radiotherapy, and chemotherapy, which can be used either alone or in combination with each other, are available [5]. Chemotherapy, a commonly used anti-cancer strategy, has been developed by screening for natural or synthetic anti-cancer compounds [4,6]. However, traditional chemotherapeutics have drawbacks, such as quick clearance, low drug tolerance due to non-specific biodistribution, and the emergence of drug resistance [3,5,7].

Photodynamic therapy (PDT) is a promising non-invasive and patient-specific anti-cancer strategy [8,9]. This therapy can be used to avoid the side effects of damage to invasive injuries caused by the systemic toxicity of chemotherapy or the proximal health tissues caused by radiotherapy. A photosensitiser (PS), which is highly tumour-retained, generates reactive oxygen species, especially singlet oxygen (^1^O_2_), when irradiated with light. The generated oxygen directly inflicts damage to, causes destruction of, and induces an immune response against targeted cells [8,9,10].

Despite their promising anti-cancer effects, many PSs are prone to aggregation owing to their lipophilic property, which causes poor bioavailability and reduced pharmacological effects [9,11]. Some approaches, such as conjugation of PSs to water-soluble polymers, colloidal administration, and encapsulation of PSs in formulations, have been reported to overcome these limitations [12,13,14]. However, considering that water-soluble polymers can easily absorb atmospheric moisture, there is a concern of the stability for formulation storage of PSs. In this regard, the limitations of water-soluble polymer-based carriers may need to be overcome [15,16].

The traditional pharmaceutical strategies in cancer therapy involve the use of enhanced permeation and retention (EPR) effect as passive targeting, as well as the use of pH-responsive biomaterials for drug delivery systems [6,17]. When a drug with anti-cancer effects is encapsulated into nano-sized carriers up to a size of 400 nm, the blood capillary permeability in the affected tissues increases with the decreased return of fluid to lymphatic circulation [2,3,18]. Therefore, the drug can efficiently accumulate in tumour sites. In the case of a pH-responsive formulation strategy, tumour cells secrete a large amount of lactic acid through hypoxic glycolysis, which causes pH dysregulation in tumour cells [1,5,19]. Hence, the formulation using acidic degraded materials can avoid damage to normal cells.

A literature search revealed that lipid-based carriers are promising candidates for improving the bioavailability of lipophilic drugs. Liposomes, a pH-responsive drug delivery system, have been widely used for cancer treatment [3,18,20]. The amphiphilic properties of phospholipids can facilitate the encapsulation of lipophilic drugs [21]. However, because of the tight bilayer structure of liposomes, flexible formulations are closer to the ideal for the EPR effect [18]. In 1992, Cevc and Blume first introduced a novel flexible liposome, named ‘transfersome’, that consists of phospholipids and an edge activator (EA) as surfactant [22]. They have been reported to penetrate intact skin [23,24,25]. The EAs destabilise the lipid bilayer, and therefore, increase the flexibility and elasticity of the lipid bilayer [26,27,28].

This study aimed to prepare and characterise PS-encapsulated nano-transfersomes for the treatment of anti-cancer effects in PDT. The synthesised PS structure was evaluated by ^1^H nuclear magnetic resonance (^1^H-NMR) spectroscopy. ^1^O_2_ photogeneration, a pharmacological effect in PDT, was determined using 1,3-diphenylisobenzofuran (DPBF) assay in both neutral and acidic environments. For the biological assay of phototoxicity, PS and PS-loaded nano-transfersomes were investigated in two tumour cell lines (HeLa and A549). Methyl pheophorbide a (MPa)-loaded nano-transfersomes exhibited burst release of drugs in an acidic environment (pH 5), which presented a drug release 4.3–7.4 times higher than that in the physiological environment (pH 7.4), which displayed sustained drug release after 24 h. This pH-responsive nano-transfersomes was affected by higher ^1^O_2_ photogeneration of MPa in an acidic environment, which presented a tumour-like environment as against the environment in neutral pH. Furthermore, the nano-transfersome system is advantageous, as it can induce greater photostability as well as enhanced photodynamic activity than free MPa. Therefore, these results suggest that our PS-loaded nano-transfersome system displaying a pH-responsive carrier is a promising anti-cancer strategy for PDT.

## 2. Materials and Methods

### 2.1. Materials

We purchased sunflower lecithin (lecithin) from Now Foods (Bloomingdale, IL, USA); chlorophyll-a paste from Shandong Lanmo Biotech Co., Ltd. (Changle, China); cholesterol, acetone, and dimethyl sulfoxide (DMSO) from Samchun (Pyeongtaek, Korea); Span^®^ 80 (SP 80), Span^®^ 20 (SP 20), and Tween^®^ 80 (TW 80) from Dae Jung Co. Ltd. (Busan, Korea); Poloxamer 407 (PX 407) from BASF (Ludwigshafen, Germany); phosphate-buffered saline (PBS), methylene blue (MB), and chloroform from Sigma-Aldrich (St. Louis, MO, USA); methylene chloride (CH_2_Cl_2_, MC) from Duksan Co. Ltd. (Gyeonggi-do, Korea); DPBF from TCI Chemical (Tokyo, Japan); Dulbecco’s modified Eagle medium from WelGENE (Gyeongsan, South Korea); and penicillin-streptomycin solution 100× and foetal bovine serum from BioWest (Nuaillé, France). Cancer cell lines (HeLa and A549) were purchased from the Korea Cell Line Bank (Seoul, Korea). The Quanti-MAX WST-8 assay kit (WST) was purchased from Biomax (Seoul, Korea). Methanol (MeOH) of high-performance liquid chromatography (HPLC) grade was purchased from Honeywell (Seelze, Germany). All other chemicals used were of HPLC grade.

### 2.2. Synthesis of MPa

MPa was synthesised via the extraction of chlorophyll-a paste. Briefly, chlorophyll-a paste (100 g) was dissolved in 5% acidic MeOH and stirred at room temperature for 12 h. The resulting solution was separated by pouring distilled water (DW). The obtained organic layer was then evaporated. The residue was purified by column chromatography using an eluent of 2% acetone/MC.

### 2.3. Preparation of MPa-Loaded Nano-Transfersomes

MPa-loaded nano-transfersomes were fabricated by sonication. Briefly, MPa and a predetermined amount of lecithin, as shown in Table 1, were dissolved in MC with/without cholesterol and EA (SP 20, SP 80, TW 80, and PX 407). The MC was removed using a rotary vacuum evaporator to form a thin lipid film. To fabricate transfersomes, the obtained thin lipid film was hydrated by pouring DW followed by sonication using a bath sonicator (K410HTD, Shenzhen Guan Yijia Technology Co., Ltd, Shenzhen, China). The prepared transfersomes were ultrasonicated using a probe sonicator (Scientz-IID, Ningbo, China) at 300 W for 15 min with a 5 s pulse-on period and a 5 s pulse-off period. The effects of different compositions of MPa-loaded nano-transfersomes were evaluated, as presented in Table 1.

### 2.4. Characterisation of MPa

#### 2.4.1. NMR Spectroscopy

All ^1^H-NMR experiments were performed using a Varian spectrometer (500 MHz, CDCl_3_) at the Biohealth Products Research Center of Inje University.

#### 2.4.2. Development of Analytical Method for MPa

The concentration of MPa was determined using a UV-Vis spectrophotometer (S-3100, Scinco, Seoul, Korea) at ambient temperature. We measured the absorption spectrum of MPa in the wavelength range of 300–800 nm to determine its maximum absorption wavelength. The solvent that demonstrated the best characteristics for this method was MeOH. A standard stock solution was prepared by dissolving 2 mg of an accurate amount of MPa into 20 mL of MeOH.

##### Linearity

The standard stock solution was diluted with MeOH to obtain a final concentration of standard as 1–20 ppm, and the five-point linearity was determined. Standard solutions of different concentrations were prepared. The calibration curves and concentration vs. absorbance units were constructed for the drugs.

##### Precision

The precision of the test method was determined by performing an assay on six replicate determinations of sample preparation at test concentrations, and the relative standard deviation (RSD) of the assay results was calculated.

##### Accuracy

To study the accuracy of the method, recovery studies were carried out by adding a known quantity of the standard to the pre-analysed sample. Recovery was performed at 0%, 25%, and 100% levels, and the contents were measured from the respective UV-Vis absorption spectra.

### 2.5. Characterisation of MPa-Loaded Nano-Transfersomes

#### 2.5.1. Determination of Nanoparticle Size, Polydispersity Index (PDI), and Zeta Potential

The particle size and PDI of the prepared nano-transfersomes were determined at 25 °C via dynamic light scattering using a Zetasizer Nano ZS (Malvern Instruments Ltd., Worcestershire, Malvern, UK). The samples were diluted 10 times with DW before measurement. For particle size, the instrument was equilibrated before each measurement. Each value reported is the average of the three measurements.

The zeta potential of the nano-transfersome was measured at 25 °C using a Zetasizer Nano ZS and estimated from the electrophoretic mobility of the particle surface. The samples were diluted 10 times with DW before measurement. The instrument was equilibrated before each measurement. Each measurement was performed in triplicate.

#### 2.5.2. Determination of Drug-Loading Capacity

The entrapment efficiency (EE) and loading amount (LA) of MPa-loaded nano-transfersomes were determined by centrifugation. Nano-transfersome preparations were diluted 10 times to a final volume of 1 mL and then gently vortexed. The suspension was then centrifuged at 1300 rpm at 4 °C for 1 h. The free drug concentration in the supernatant was analysed using a UV-Vis spectrophotometer, as described in Section 2.4.2. EE and LA were calculated using Equations (1) and (2), respectively:

(1) $$\mathrm{EE}\;\left(\%\right)=\frac{\mathrm{Amount}\;\mathrm{of}\;\mathrm{total}\;\mathrm{drug}\;\mathrm{content}-\mathrm{Amount}\;\mathrm{of}\;\mathrm{free}\;\mathrm{drug}}{\mathrm{Amount}\;\mathrm{of}\;\mathrm{total}\;\mathrm{drug}\;\mathrm{content}}\times 100$$

(2) $$\mathrm{LA}\;\left(\%\right)=\frac{\mathrm{Amount}\;\mathrm{of}\;\mathrm{total}\;\mathrm{drug}\;\mathrm{content}-\mathrm{Amount}\;\mathrm{of}\;\mathrm{free}\;\mathrm{drug}}{\left(\mathrm{Amount}\;\mathrm{of}\;\mathrm{total}\;\mathrm{drug}\;\mathrm{content}-\mathrm{Amount}\;\mathrm{of}\;\mathrm{free}\;\mathrm{drug}\right)+\mathrm{Amount}\;\mathrm{of}\;\mathrm{lipid}}\times 100$$

#### 2.5.3. Fourier Transform Infrared Attenuated Total Reflection (FTIR-ATR) Spectroscopy

To obtain direct information about the chemical interaction between MPa and transfersome ingredients, FTIR spectroscopy was performed using an FTIR-ATR spectrometer (Spectrum Two FT-IR Spectrometer, PerkinElmer, Waltham, MA, USA) equipped with a ZnSe crystal. The spectra were recorded over the wavenumber range of 4000–800 cm^−1^.

### 2.6. In Vitro MPa-Release Studies

An in vitro MPa release study was performed using the dialysis bag method. Dialysis bags (Spectrum Laboratories, Inc., Compton, CA, USA) with a molecular weight of 10 kDa were soaked in DW for 12 h before the experiment. A predetermined amount of each test substance was soaked in dialysis bags, and both ends were sealed using a string. Dialysis bags were immersed in 70 mL vials containing 50 mL of receptor medium (PBS pH 7.4 or pH 5). The vials were then placed in a shaking incubator (JSSI-100T, JS Research Inc., Gongju, Korea) and shaken at 100 rpm at 37 ± 0.5 °C. At predetermined time intervals (1, 2, 4, 8, 12, 24, and 48 h), aliquots of 1 mL were withdrawn from the vial, passed through 0.45 μm membrane filters (SFCA Syringe Filters, Corning Inc., Hampto, VA, USA), and immediately analysed using a UV-Vis spectrophotometer, as described in Section 2.4.2.

### 2.7. Photo-Stability Studies

The photostability of MPa in nano-transfersomes was determined by comparison with MPa in a 0.1% MeOH solution, according to the modified method described by Lima et al. [29]. The photostability of MPa was monitored by recording its absorption spectrum at a wavelength of 664 nm. Briefly, 20 mL of MPa or MPa-loaded nano-transfersomes in a 0.1% MeOH solution (4.0 μg/mL) was irradiated (2 J/cm^2^) with LED at different time intervals (0, 10, 20, 30, and 40 min). MPa was then extracted from the transfersomes by adding 1 mL of hexane to melt the lipids, followed by vortexing. A 0.1% MeOH layer containing the extracted MPa was filtered through 0.22 μm filters, and the UV-Vis spectrophotometer was measured as described in Section 2.4.2.

### 2.8. ^1^O_2_ Photogeneration

^1^O_2_ photogeneration study was determined using DPBF. DPBF, a selective ^1^O_2_ acceptor, is bleached upon reaction with ^1^O_2_, which leads to a decrease in the intensity of the DPBF absorption band. Each sample (1 μM) with 50 μM of DPBF in DMSO was used to evaluate photogeneration. The negative control (NC) and positive control (PC) were 50 μM DPBF and 1 μM MB with 50 μM DPBF, respectively. All samples prepared in the dark were placed in a 48-well plate and covered with aluminium foil. The plate was irradiated (2 J/cm^2^) with an LED for 15 min. The absorbance of each sample was measured at 418 nm using a microplate reader (Synergy HTX, BioTek, Winooski, VT, USA).

### 2.9. In Vitro Photo-Irritation Studies

#### 2.9.1. Cytotoxicity Study Using Tumour Cell from Human

To evaluate the anti-cancer efficacy after photoirradiation, we investigated the cytotoxic effects of each component of the nano-transfersomes in tumour cell lines. Two cell lines (HeLa from human cervical carcinoma and A549 from human lung carcinoma) were seeded into 48-well plates at 2 × 10^4^ cells/well, and the number of cells was calculated using a haemocytometer. Prior to each experiment, the cells were incubated for 24 h at 37 ± 0.5 °C in a humidified atmosphere with 5% CO_2_. Then, various concentrations (1, 2.5, 5, and 10 μM) of each sample were added to each well. After 24 h, the exposed cells were rinsed with sterile PBS, and 200 µL/well of the growth medium was added. The cells were then irradiated (2 J/cm^2^) with an LED at a distance of 20 cm for 15 min. The treated cells were incubated for 24 h at 37 ± 0.5 °C and 5% CO_2_ for the WST reduction experiment as described in Section 2.9.2.

#### 2.9.2. Viability of Cancer Cells

Cytotoxicity was determined by measuring the dehydrogenase activity of viable keratinocytes 24 h after incubation. The activity was determined after the incorporation of WST as described previously [30]. Each cell line was treated with 100 µL/well of 10% WST solution for 1 h. The concentration of WST was measured by determining the optical density (OD) at 450 nm using a microplate reader.

Each experiment was conducted in at least three wells of a well plate. After subtracting the blank OD from all raw data, the mean OD values ± standard deviations (SDs) were calculated using three measurements per test substance, and the percentage of cell viability was expressed using Equation (3) relative to that of the NC. The NC value was set at 100%:

(3) $$\mathrm{Viability}\;\left(\%\right)=\frac{{\mathrm{Mean}\;\mathrm{OD}}_{\mathrm{treated}}}{{\mathrm{Mean}\;\mathrm{OD}}_{\mathrm{control}}}\times 100$$

### 2.10. Statistical Analysis

Three independent experiments were conducted for all analyses. The presented data (mean ± SD) were compared using one-way analysis of variance and Student’s *t*-tests. Statistical significance was set at *p* < 0.05.

## 3. Results and Discussion

### 3.1. Characterisation of MPa

#### 3.1.1. NMR Spectroscopy

**Figure 1:**
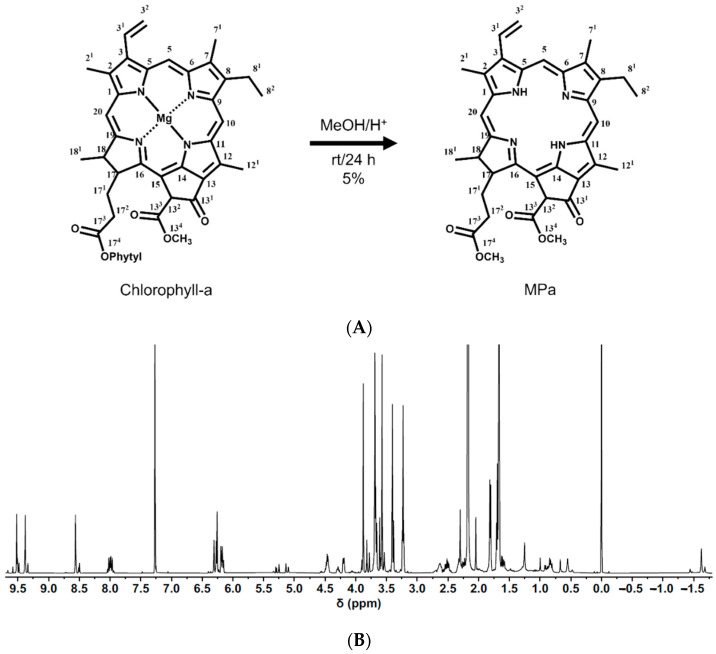
(**A**) Synthetic scheme of MPa from chlorophyll-a with numbering, (**B**) ^1^H-NMR spectrum of MPa (500 MHz, CDCl_3_, 25 °C, TMS).

The structure of MPa was characterised by ^1^H-NMR spectroscopy. Figure 1 shows the ^1^H-NMR spectrum of MPa. ^1^H-NMR (500 MHz, CDCl_3_, 25 °C, TMS): δ 9.52 (s, 1H, 10H), 9.38 (s, 1H, 5H), 8.56 (s, 1H, 20H), 8.04–7.94 (m, 1H, 3^1^H), 6.31–6.15 (m, 2H, 3^2^H), 5.25 (m, 1H, 13^2^H), 4.47 (m, 1H, 17H), 4.21 (m, 1H, 18H), 3.88 (s, 3H, OCH_3_, 13^4^H), 3.69 (s, 3H, 12^1^H), 3.68 (q, J = 8 Hz, 2H, 8^1^H), 3.57 (s, 3H, OCH_3_, 17^4^H), 3.40 (s, 3H, 2^1^H), 3.23 (m, 3H, 7^1^H), 2.63 (m, 1H, 17^2^H), 2.52–2.32 (m, 2H, 17^1^H), 2.23 (m, 1H, 17^2^H), 1.82 (d, J = 7 Hz, 3H, 18^1^H), 1.69 (t, 3H, 8^2^H, overlapped with solvent), and 0.55 and −1.62 (all brs and each 1H, NH). Our results revealed that the peak for 17^4^H (OCH_3_), 3.57 ppm, is due to the elimination of the phytyl group in the chlorophyll-a paste. Regarding the peaks for NH, 0.55 and −1.62 ppm, Mg^2+^ in the MPa macrocyclic ring system was degraded during MPa synthesis process.

#### 3.1.2. Development of Analytical Method for MPa

##### The Absorption Spectra and Specificity of MPa

**Figure 2:**
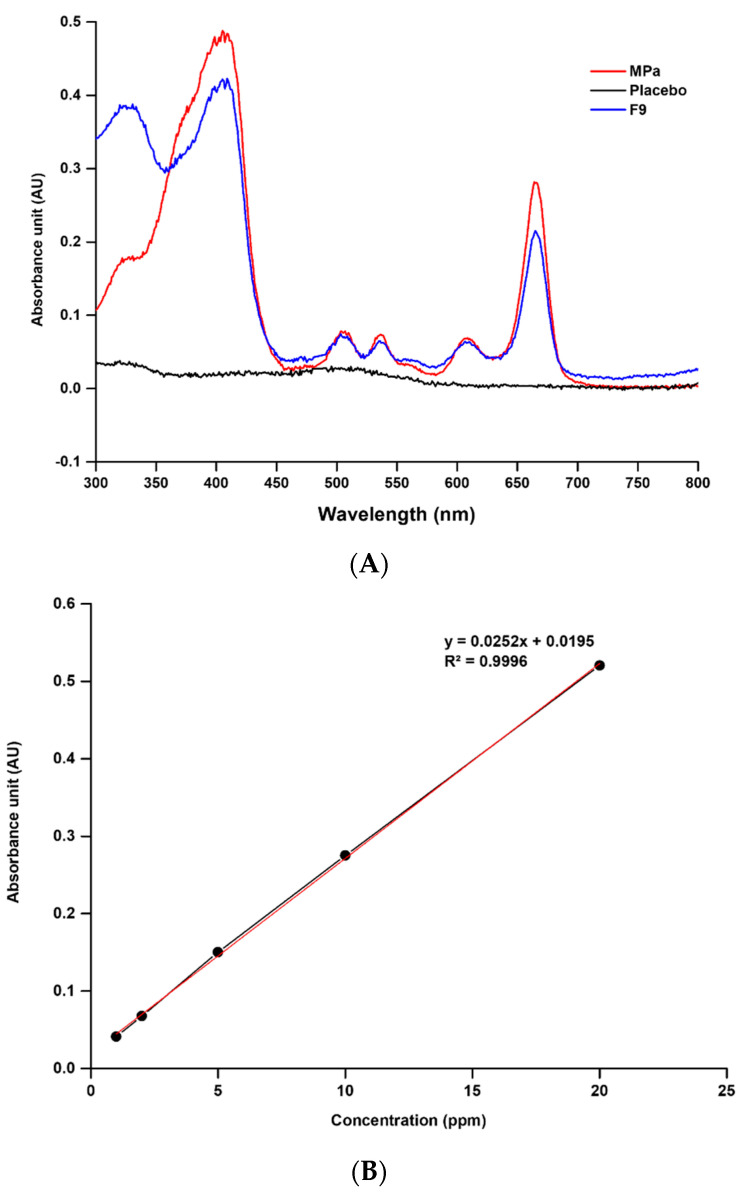
UV-Vis spectra and calibration curve of MPa. (**A**) Specificity data of MPa, placebo, and MPa-loaded nano-transfersome F9 (MeOH, 25 °C). (**B**) Linearity data of MPa standard stock solution in MeOH.

We evaluated the absorption spectra and specificity of MPa to determine the maximum absorption wavelength and to identify the specific absorption spectra [31,32,33]. The UV-Vis spectra showed that the maximum absorption wavelength was 664 nm, as shown in Figure 2. In addition, the UV-Vis spectra of the placebo transfersome indicated that there was no interference of the placebo with the analyte. Therefore, we analysed MPa at 664 nm.

##### Linearity

A calibration curve was prepared by analysing five standard solutions with concentrations in the range of 1–20 ppm for MPa. The correlation coefficient of the calibration curve determined by linear regression analysis was 0.9996, as shown in Figure 2.

##### Precision

Precision defined the closeness of agreement among measurements from different samples of the same concentration of standard stock solution. Precision was expressed as the RSD of repeatability. Thus, the parameters were calculated as RSD (%) of the absorption unit. The precision results demonstrated that the RSD (%) value of the recovery was 1.56%, as shown in Table 2. This result indicates the high precision of the proposed analytical method.

##### Accuracy

We also estimated the accuracy of the developed analysis method. Accuracy is defined as the closeness of agreement between the test results and a conventional true or accepted reference value. Accuracy was expressed as the RSD calculated from the drug recovery. The accuracy results for the RSD (%) values of the recovery were 0.96, 0.19, and 0.17%, respectively, as shown in Table 3. This result reveals the accuracy of the developed analytical method.

### 3.2. Characterisation of MPa-Loaded Nano-Transfersomes

#### 3.2.1. Nanoparticle Size, PDI, and Zeta Potential

**Figure 3:**
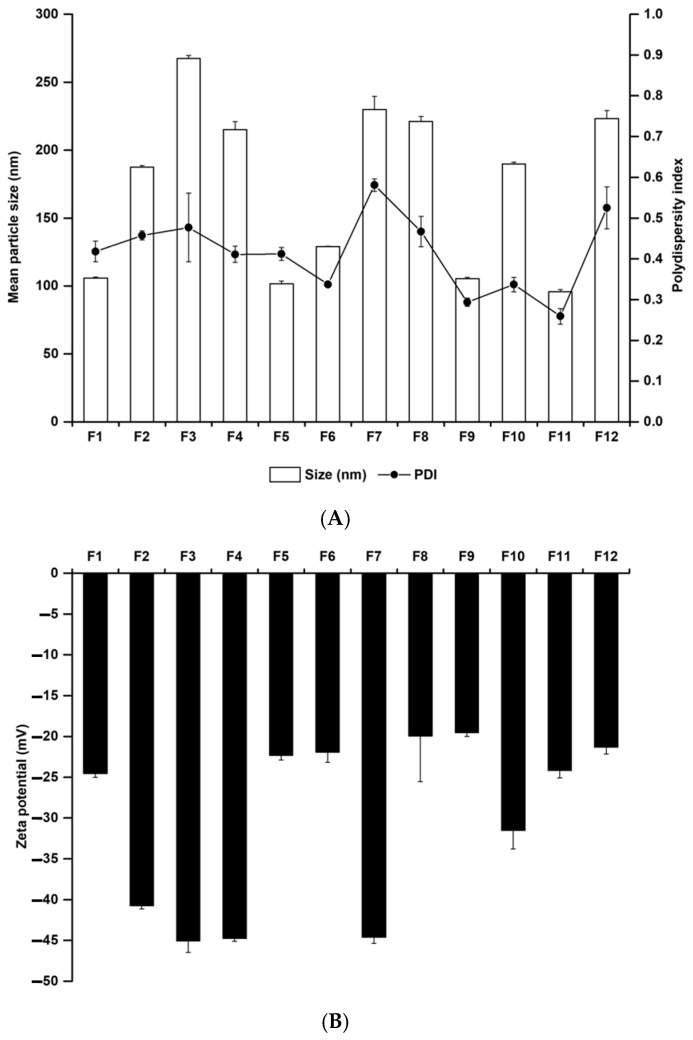
Physicochemical characteristics of (**A**) average particle size and PDI and (**B**) zeta potential for MPa-loaded nano-transfersomes prepared using different materials. Results are expressed as the means ± standard deviations of three independent experiments (*n* = 3). MPa, methyl pheophorbide-a; PDI, polydispersity index.

Particle characterisation studies were carried out to evaluate the effects of various materials, including membrane stabilisers and EAs on the formation of nanoparticles. Particle size plays an important role in cancer therapy because of passive targeting based on the EPR effect [17,34]. Zeta potential demonstrates the degree of nanoparticle aggregation, which is an important factor in storage stability. With a high energy barrier on the surface of nanoparticles, a high zeta potential ensures high storage stability, whereas a low zeta potential facilitates the release of encapsulated drugs [35]. Thus, moderate zeta potential is important for the balance between drug release and storage stability of the formulation. Figure 3 shows the particle size, PDI, and zeta potential of the MPa-loaded nano-transfersomes. Liposome F2 containing cholesterol had higher particle size and zeta potential than liposome F1 with no cholesterol. This suggests that cholesterol provides rigidity to the phospholipid bilayer of liposomes, which hinders the sonication energy transition during the probe sonication process [21,36]. In addition, the higher zeta potential of liposomes was attributed to the rigid phospholipid bilayer, which caused the exposure of lipid head groups.

We evaluated the effect of EA on particle size and zeta potential. Lower particle sizes and zeta potentials were obtained by decreasing the lipophilicity of lipophilic surfactants (SP 80 and SP 20) or hydrophilicity of hydrophilic surfactants (TW 80 and PX 407). The hydrophilic-lipophilic balance (HLB) determines the hydrophilicity and lipophilicity of surfactants, and the HLB values of SP 80, SP 20, TW 80, and PX 407 are 4.3, 8.6, 15, and 22, respectively [37,38,39]. The effect of lipophilic surfactants on the increase in particle size was stronger than that of hydrophilic surfactants. This could be explained by the fact that hydrophilic surfactants are incorporated into inner/outer aqueous phases, whereas lipophilic surfactants are incorporated into the lipid bilayer [26,27,28].

#### 3.2.2. Determination of the Drug-Loading Capacity

**Figure 4:**
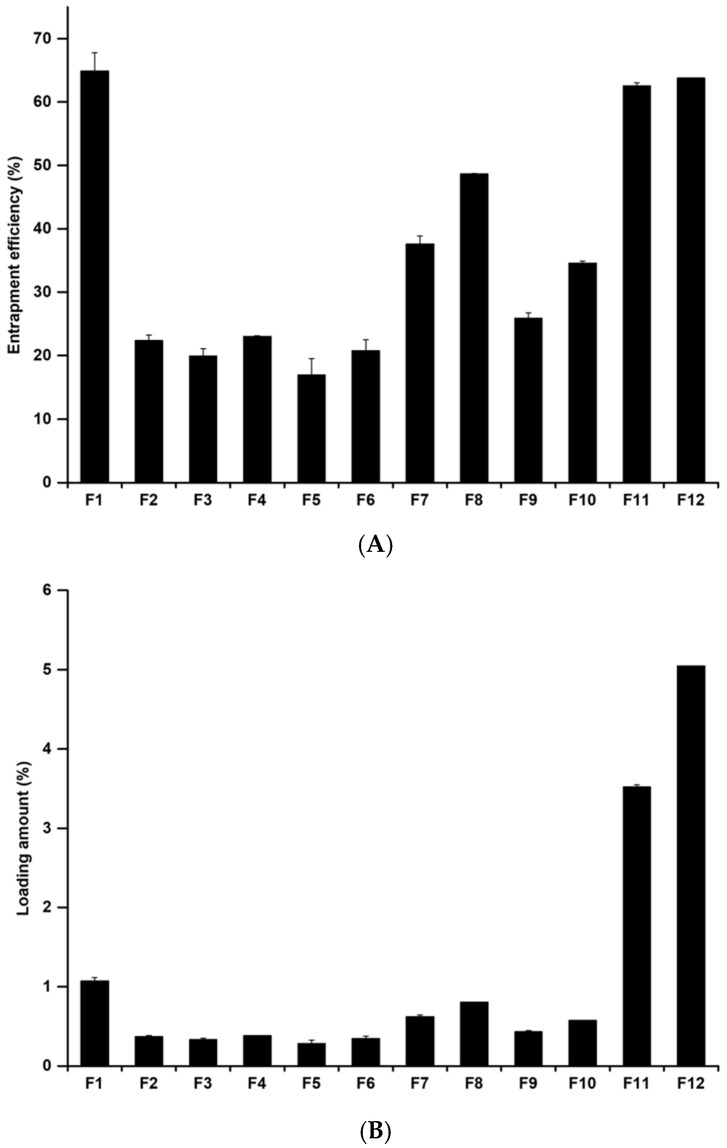
(**A**) Entrapment efficiency and (**B**) loading amount of MPa-loaded nano-transfersomes prepared by using different compositions. Results are expressed as the means ± standard deviations of three independent experiments (*n* = 3). MPa, methyl pheophorbide-a.

The loading capacity (EE and LA) is an important parameter in drug delivery systems because it improves the photostability of MPa, enables sustained release of MPa from nano-transfersomes, and avoids side effects. We evaluated the EE and LA of MPa-loaded nano-transfersomes using the centrifugation method, followed by the estimation of MPa using the UV-Vis method. Figure 4 shows the EE and LA of the MPa-loaded nano-transfersomes. Our results revealed that the EE and LA of the formulations range from 16.95 ± 2.60% to 64.87 ± 2.93% and 0.28 ± 0.04% to 5.05 ± 0.00%, respectively. The loading capacity of the formulations with cholesterol (F7–F10) was higher than that without cholesterol (F3–F6). This suggests that cholesterol is a lipid that increases the volume of the lipid bilayer [25,26,28]. Therefore, MPa, a hydrophobic drug, was more encapsulated in transfersomes with cholesterol than in those without cholesterol.

Considering the effects of EA, the loading capacities of F3, F4, F7, and F8 (formulations using lipophilic surfactants) were higher than those of F5, F6, F9, and F10 (formulations using hydrophilic surfactants). This suggests that the volume of the lipid bilayer encapsulating MPa increases, as mentioned above, due to the effect of cholesterol on the loading capacity. In addition, the loading capacity of the formulations using surfactants with higher lipophilicity tended to decrease. This suggests that EA is used to improve the deformability of vesicles by forming pores in the lipid bilayers [27,28].

#### 3.2.3. FTIR-ATR Spectroscopy

**Figure 5:**
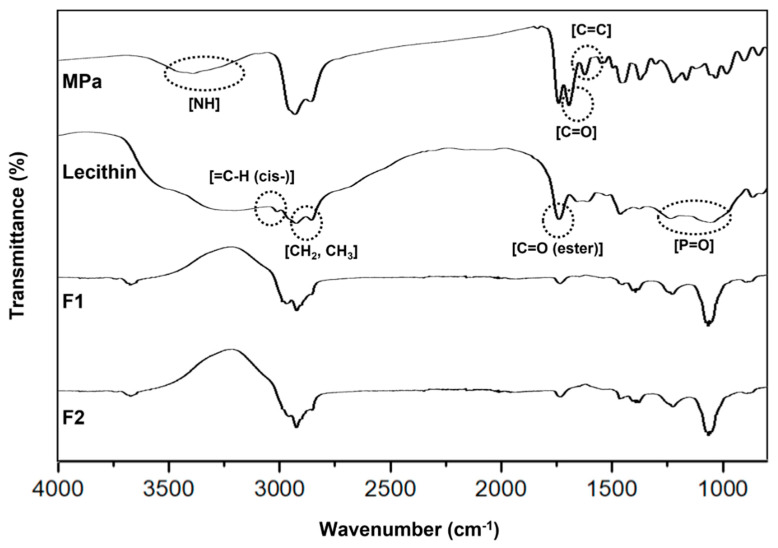
Fourier transform infrared spectroscopy (FTIR) overlay spectra of transfersomes. Pure MPa; lecithin; F1: transfersome consisting of MPa and lecithin; F2: transfersome consisting of MPa, lecithin, and cholesterol.

We performed FTIR-ATR spectroscopy to determine the chemical interactions between MPa and the ingredients of the transfersomes, for which we selected to use F1 and F2 of the formulations because they are the main components of our transfersome. Figure 5 shows the FTIR spectra of MPa, lecithin, F1, and F2. The FTIR spectrum of the synthesised MPa exhibited characteristic peaks at 1622 cm^−1^ (C=C stretching), 1693 cm^−1^ (C=O stretching), and 3393 cm^−1^ (N-H stretching), as summarised in Table 4. The FTIR spectra of F1 and F2 demonstrated that the peaks for both formulations were not detected for both C=C stretching and C=O stretching and shifted for N-H stretching to 3675 and 3672 cm^−1^, respectively. This suggests that cholesterol is the difference in composition between F1 and F2 [40,41]. Additionally, the peak of N-H stretching in MPa was slightly shifted when comparing F1 with F2. Therefore, the H of N-H stretching in MPa forms a weak H-bond (hydrogen bond) with the hydroxyl group in cholesterol [40,41].

Figure 5 also shows that the FTIR spectrum of lecithin demonstrated characteristic peaks at 2925 and 2855 cm^−1^ (CH_2_, CH_3_ stretching), 3010 cm^−1^ (=C-H (cis-) stretching in monounsaturated fatty acid part of the lecithin structure), 1738 cm^−1^ (C=O stretching as part of the ester in the lecithin structure), and 1235 and 1055 cm^−1^ (P=O stretching), as summarised in Table 4. The FTIR spectrum of F1 showed that there were slight changes in the fingerprint region, that is, the transmittance peaks of CH_2_ and CH_3_ stretching to 2923 and 2855 cm^−1^, the =C-H (cis-) stretching peak was absent, the C=O (ester) stretching peak at 1735 cm^−1^, and the P=O stretching peak to 1229 and 1066 cm^−1^. The FTIR spectrum of F2 revealed that CH_2_ and CH_3_ stretching shifted to 2924 and 2853 cm^−1^; the =C-H (cis-) stretching peak was not detected; the C=O (ester) stretching peak at 1735 cm^−1^; and the P=O stretching peak to 1229 and 1066 cm^−1^. The peak shift of CH_2_ and CH_3_ for F2 compared with that for F1 might be attributed to van der Waals interactions between the CH_2_ and CH_3_ peaks of the lecithin structure [42]. In addition, the difference in composition between F1 and F2 was the presence of cholesterol.

Regarding the chemical interactions between MPa and lecithin, MPa was bonded with lecithin via three points of H-bonding (the interactions between C=O of MPa and =C-H (cis-) of lecithin, NH of MPa and P=O of lecithin, and NH of MPa and C=O (ester)) and one point of van der Waals forces (the interaction between C=C of MPa and CH_2_, CH_3_ of lecithin). The characteristic peak of NH stretching for MPa was shifted to 3657 cm^−1^ with the shifted peaks of C=O (ester) stretching to 1735 cm^−1^ and P=O stretching at 1229 cm^−1^ and 1066 cm^−1^ for lecithin. In addition, considering that the peak of the C=O (ester) stretching peak was slightly changed, unlike that of P=O stretching, the H-bond between the P=O of lecithin and the NH of MPa may be strong. In the case of the H-bond between C=O of MPa and =C-H (cis-) of lecithin, the =C-H (cis-) peak of the monounsaturated fatty acid in lecithin was absent among the potential H-bond sites [42]. The possible sites involving van der Waals forces in lecithin are the CH_2_ and CH_3_ groups of the monounsaturated and saturated fatty acids, and those in MPa are C=C. The peaks of C=C of MPa and CH_2_ and CH_3_ of lecithin disappeared and shifted in F1 and F2, respectively.

### 3.3. In Vitro MPa-Release Studies

**Figure 6:**
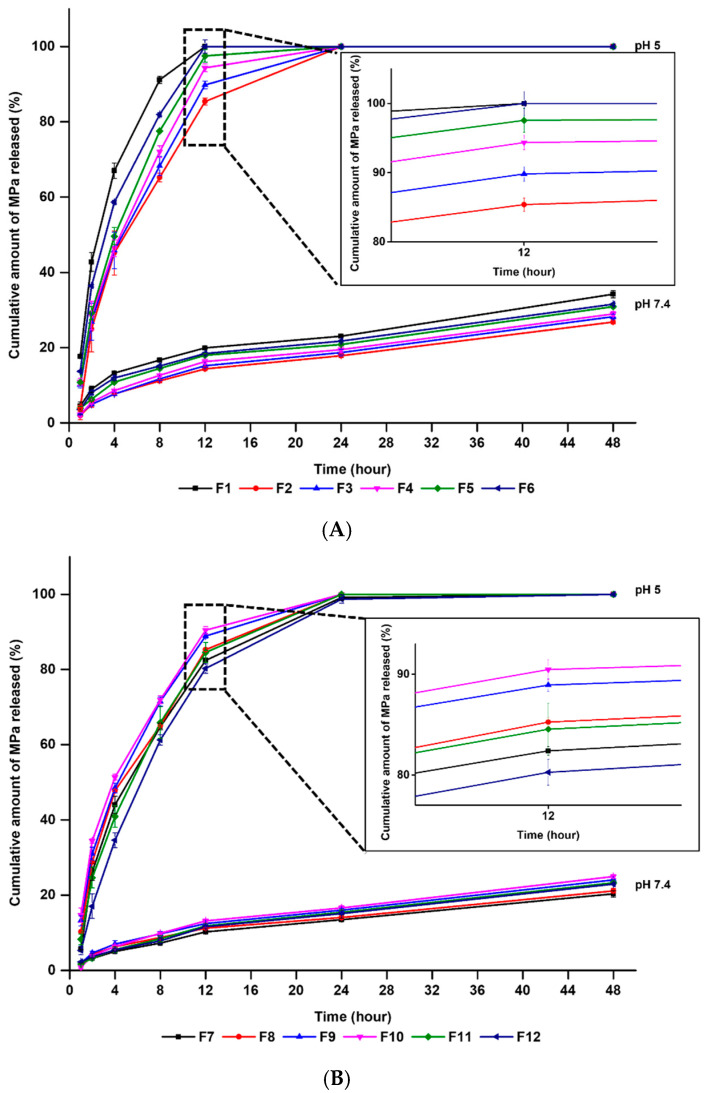
Cumulative percentage release profiles of methyl pheophorbide-a from nano-transfersomes that are (A) F1–F6 and (B) F7–F12 in release medium (pH 7.4 or pH 5), as determined using dialysis bag method. Results are expressed as the means ± standard errors of three independent experiments (*n* = 3).

The in vitro release profiles of the MPa-loaded nano-transfersomes were obtained using the dialysis membrane method. In particular, we carried out the drug release profiles in both pH 7.4 and pH 5, to simulate the human body and cancer cell environments, respectively. The results of MPa release from the formulations in pH 7.4 ranged from 13.44 to 23.02% and from 20.31 to 34.20% after 24 h and 48 h, respectively, whereas those in pH 5 were approximately 100% after 24 h (4.3–7.4 times higher than that in pH 7.4), as shown in Figure 6. Additionally, the MPa release results of all formulations in the receptor medium at pH 7.4 demonstrated sustained release, whereas that at pH 5 of receptor medium showed burst release. This result might be attributed to lecithin, which is a pH-responsive phospholipid that is easily degraded in acidic environments [20]. Thus, MPa encapsulated in nano-transfersomes not only might be delivered into cancer cells without leakage in the body, but also was rapidly released from nano-transfersomes in cancer cell, which induce an enhanced PDT effect.

Regarding the effects of EA, an increase in lipophilicity of the surfactant resulted in a delayed release. The order of the release amount of MPa after 48 h was F1 > F6 > F5 > F4 > F3 > F2 > F10 > F9 > F11 > F12 > F8 > F7. The results of MPa release demonstrated that F3 and F7 using the surfactant with the highest lipophilicity were delayed compared to F6 and F10 using the surfactant with the lowest lipophilicity. The reason for this might be the affinity between the lipophilic surfactant and MPa with its lipophilic property [27,28]. In this regard, the MPa release from F7, F8, F9, and F10 (formulations using cholesterol) were delayed compared to F3, F4, F5, and F6 (formulations without cholesterol).

### 3.4. Photo-Stability Studies

**Figure 7:**
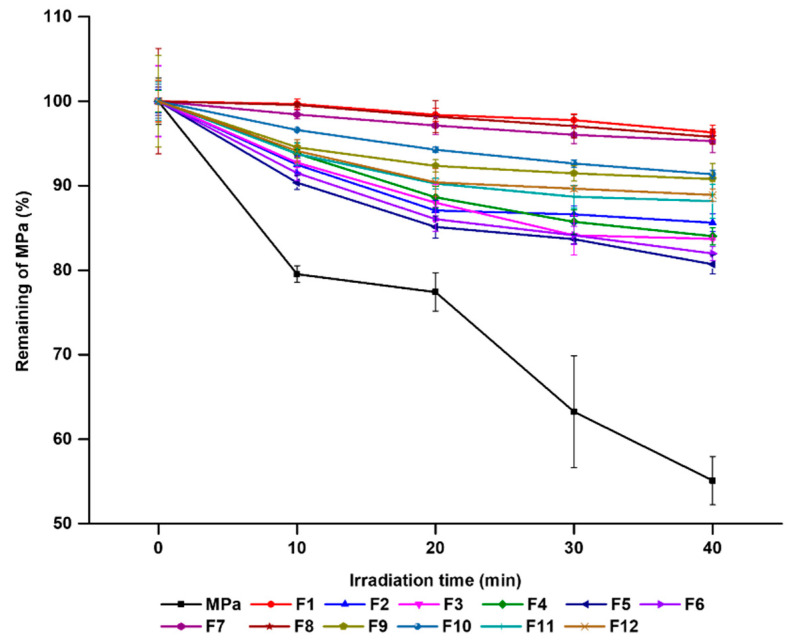
Photostability test using percentage of non-degraded MPa from free MPa solution and MPa-loaded nano-transfersomes before and after irradiation with LED of 2 J/cm^2^ for different time intervals of 0, 10, 20, 30, and 40 min. Results are expressed as means ± standard deviations of three independent experiments (*n* = 3).

The photostability of PS plays an important role in the light-induced therapeutic capacities. In this study, the photostability of MPa in MPa-loaded nano-transfersomes and MPa solutions was compared. Figure 7 presents the remaining MPa with/without the transfersomes. All transfersomes caused greater photostability of MPa than the MPa solution, which were 80.69–96.32% and 55.09% for 40 min irradiation, respectively. This suggests that the structure of transfersomes can physically cut off from the external environment, and therefore, the stability of MPa encapsulated in transfersomes was improved [25,43].

Concerning the effect of EA, MPa, and MPa-loaded nano-transfersomes showed the following order of photostability after 40 min of irradiation: F1 > F8 > F7 > F10 > F9 > F12 > F11 > F2 > F4 > F3 > F6 > F5 > free MPa solution, which demonstrated that the formulations using the lipophilic surfactant (F3, F4, F7, and F8) had higher amounts of MPa than those using hydrophilic surfactants (F5, F6, F9, and F10). In addition, the formulations using relatively high lipophilic surfactants were unstable. This result might be attributed to the relatively high loading capacity, as shown in Figure 4 [27,28]. An increase in the loaded amount of MPa in the transfersomes increased the light-protected MPa, as mentioned above.

### 3.5. ^1^O_2_ Photogeneration

**Figure 8:**
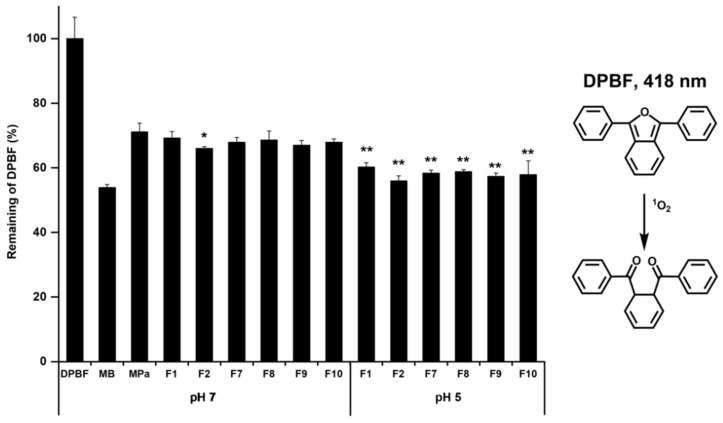
DPBF (50 μM in DMSO) absorbance decay (%) for the ^1^O_2_ photogeneration efficacy of MPa with/without transfersome in pH 7 or pH 5 at 418 nm after photoirradiation (total light dose 2 J/cm^2^; irradiation time 15 min). Results are expressed as means ± standard deviations of three independent experiments (*n* = 3). NC: DPBF (1,3-diphenylisobenzofuran); PC: MB (methylene blue); MPa (methyl pheophorbide-a). Statistical significance of the difference in DPBF between free MPa solution and the formulations is indicated by either a single asterisk (*p* < 0.05) or double asterisks (*p* < 0.01).

We evaluated the ^1^O_2_ photogeneration using the DPBF assay to indirectly determine the pharmacological effects of MPa. DPBF reacts with ^1^O_2_, which was created after photoirradiation of MPa. Consequently, the intensity of the DPBF absorption band decreases. MB, a standard ^1^O_2_ sensitiser, was used to compare the pharmacological effects of MPa. The results of the DPBF assay demonstrated that the pharmacological effects of MPa with/without transfersomes were lower than those of MB, indicating that the intensity of the remained DPBF was high, as shown in Figure 8. Concerning the pharmacological effect of MPa, the intensity values of the remained DPBF for free MPa and MPa-loaded nano-transfersomes were 71.10% and 55.95–69.25%, respectively. This result indicates that the pharmacological effects of MPa were enhanced when MPa was encapsulated into the transfersomes. The reason for this might be that the aggregation of MPa was inhibited by incorporation into the transfersomes. Concerning the pH environment, the intensity values of the remained DPBF for the formulations in the acidic environment (pH 5, average value 58.1%) were lower than those in the neutral environment (pH 7, average value 67.8%). This suggests that lecithin is degradable in acidic environments and leads to enhanced MPa release, indicating that lecithin-based nano-transfersomes of MPa are pH-responsive formulations with improved PDT effects [20].

Regarding the EAs, the intensity values of the remained DPBF for the formulations using hydrophilic surfactant were lower than those using a lipophilic surfactant, which was in both neutral and acidic environments (remained DPFE average values of the formulations containing hydrophilic EAs or lipophilic EAs: 67.5% and 68.3% at pH 7, and 57.7% and 58.6% at pH 5, respectively). This suggests that the formulations using a lipophilic surfactant had a relatively high loading capacity when compared with that using a hydrophilic surfactant. Therefore, the encapsulated MPa was protected from light. In the case of the HLB values, the formulations using hydrophilic or lipophilic surfactants with relatively high lipophilicity demonstrated low-intensity values of the remained DPBF. The reason for this might be that the phospholipid bilayer was deformed by the EA, as mentioned above, in the drug-loading capacity [27,28].

### 3.6. In Vitro Photo-Irritation Studies

**Figure 9:**
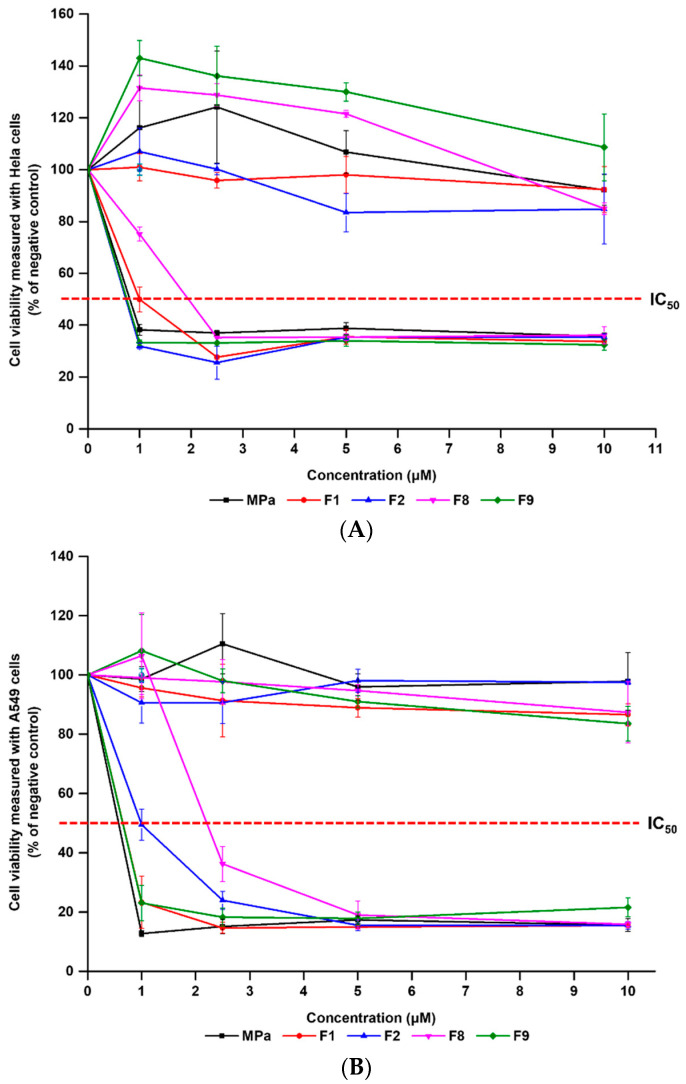
Viability of two cancer cell lines (HeLa and A549) treated with free MPa solution, F1, F2, F8, and F9. The cell viability was measured using WST assay. (**A**) Dark and light cytotoxicity of HeLa cells, (**B**) dark and light cytotoxicity of A549 cells. Results are expressed as means ± standard deviations of three independent experiments (*n* = 3).

We investigated the cytotoxic effects of MPa against two cancer cell lines (HeLa and A549). The cytotoxicity of MPa was estimated using the WST assay, as shown in Figure 9 and Supplementary Table S1–S4. A dark cytotoxicity assay was performed to determine the cytotoxic effects of the normal state of the formulations. However, light cytotoxicity, as a photo-irritation, was performed to determine the pharmacological effect. F1, F2, F8, and F9 were used to evaluate the anti-cancer effects. F1 and F2 were prepared using lecithin with/without cholesterol, which are the main components of transfersome. F8 was fabricated using a lipophilic surfactant (SP 20) with a relatively large particle size (221.1 nm) and high EE (48.7%), whereas F9 used a hydrophilic surfactant (TW 80) with a relatively small particle size (105.4 nm) and low EE (25.9%). In particular, we used various concentrations of each sample (1, 2.5, 5, and 10 μM) to estimate the inhibitory medium concentration values (IC_50_). The results of dark cytotoxicity demonstrated that all test substances were non-irritating substances against both HeLa and A549 cells, which were 83.41–143.01% and 83.58–110.53%, respectively. Therefore, the normal state of the nano-transfersomes had negligible dark toxicity for cancer cells.

Upon photoirradiation followed by 24 h after incubation, all nano-transfersomes showed decreased cell viability, which was dependent on the cell type (HeLa and A549) and concentration of the PS. Based on the IC_50_ values (Table 5), the order of PDT activity was as follows: F2 (0.73 μM) ≈ F9 (0.75 μM) > MPa (0.81 μM) > F1 (1.00 μM) > F8 (1.95 μM) in HeLa cells; MPa (0.57 μM) > F1 = F9 (0.65 μM) > F2 (0.99 μM) > F8 (2.21 μM) in A549 cells. In HeLa cells, nano-transfersomes F2 and F9 displayed enhanced PDT activity (IC_50_, 0.73 and 0.75 μM, respectively) compared with free MPa (IC_50_, 0.81 μM). These results indicate that the nano-transfersomes F2 and F9 did not prevent the original PDT activity of MPa after the formation of nano-transfersomes, but rather increased it. On the other hand, in A549 cells, nano-transfersomes F1 and F9 slightly decreased PDT activity of free MPa (IC_50_, 0.65, 0.65, and 0.57 μM, respectively). Interestingly, in both HeLa and A549 cells, F9 containing hydrophilic EA (TW 80) presented better PDT activity than F8 containing hydrophobic EA (SP 20), which is consistent with better ^1^O_2_ photogeneration of F9 than F8 (Figure 8). This might be due to the hydrophilic EA resulting in smaller particle size than the hydrophobic EA, which may result in better cellular uptake based on the size effect. Therefore, it is noted that the size effect is more important than the loading capacity for the anti-cancer effect. Furthermore, as a membrane stabiliser, rigid liposome F2 containing cholesterol presents cell type-dependent results when compared with flexible liposome F1 without cholesterol (F2, 0.73 and F1, 1.00 μM in HeLa; F2, 0.99 and F1, 0.65 μM in A549). Usually, there is a delayed release in rigid liposomes compared to flexible liposomes; however, these results indicate that there was a negligible rigidity effect inducing delayed release from the liposome [9,12,13,19,20].

## 4. Conclusions

The objective of this study was to synthesise and prepare PS and PS-loaded nano-transfersomes for the treatment of anti-cancer effects in PDT. The ^1^H-NMR results confirmed that the phytyl group in chlorophyll-a was eliminated, and NH in MPa was formed. The FTIR-ATR results of PS-loaded nano-transfersomes demonstrated that MPa interacted with lecithin by both H-bond and van der Waals forces, leading to the encapsulation of PS into the nano-transfersomes. The results of drug release showed sustained drug release in the physiological environment, but burst release in an acidic environment, demonstrating that our lecithin-based nano-transfersome is not only effectively delivered into the cancer cells without leakage in the body but also shows a pH-responsive release to enhanced photodynamic activity in cancer cells. The photo-irritation studies using two cancer cell lines (HeLa and A549) confirmed that the formulations had no dark toxicity but displayed light toxicity. This means that in the normal state, the formulations had no effect on cancer cells, but with light irradiation, they showed anti-cancer effects. In particular, our photo-irritation results revealed that the anti-cancer effect is dominated by particle size rather than loading capacity. Therefore, these results suggest that our PS-loaded nano-transfersome system is a promising anti-cancer strategy for PDT.

## Figures and Tables

**Table 1:** Composition of MPa-loaded nano-transfersomes.

	Drug (mg)	Lipid (mg)	Membrane Stabiliser (mg)	Edge Activator (mg)			
	MPa	Lecithin	Cholesterol	SP 80	SP 20	TW 80	PX 407
F1	10	600					
F2	10	600	200				
F3	10	600		200			
F4	10	600			200		
F5	10	600				200	
F6	10	600					200
F7	10	600	200	200			
F8	10	600	200		200		
F9	10	600	200			200	
F10	10	600	200				200
F11	30	600	200			200	
F12	50	600	200			200	

MPa, methyl pheophorbide-a; SP 80, Span^®^ 80; SP 20, Span^®^ 20; TW 80, Tween^®^ 80; PX 407, Poloxamer 407.

**Table 2:** Precision data obtained from the developed analysis of MPa.

No.	Recovery (%)
1	103.89
2	100.33
3	100.49
4	102.85
5	104.16
6	103.92
Average (%)	102.61
SD (%)	1.61
RSD (%)	1.56

**Table 3:** Accuracy data obtained from the developed analysis of MPa.

Drug (ppm)	No	Recovery (%)	Average (%)	SD (%)	RSD (%)
1	1	110.84	109.43	1.06	0.96
	2	108.32			
	3	109.12			
5	1	100.00	100.08	0.19	0.19
	2	99.90			
	3	100.33			
20	1	100.00	100.14	0.17	0.17
	2	100.05			
	3	100.38			

**Table 4:** Infrared absorption (cm^−1^) of both C=C; C=O; and NH stretching in MPa, F1, and F2 and CH_2_, CH_3_; =C-H (cis-); C=O (ester); and P=O stretching in lecithin, F1, and F2.

	C=C (cm^−1^)	C=O (cm^−1^)	NH (cm^−1^)	CH_2_, CH_3_ (cm^−1^)	=C–H (cis-) (cm^−1^)	C=O (ester) (cm^−1^)	P=O (cm^−1^)
MPa	1622	1693	3393	-	-	-	-
Lecithin	-	-	-	2925, 2855	3010	1738	1235, 1055
F1	-	-	3675	2923, 2855	-	1735	1229, 1066
F2	-	-	3672	2924, 2853	-	1735	1229, 1066

**Table 5:** IC_50_ (μM) values against HeLa or A549 cells, particle size, and entrapment efficiency (EE) of free MPa solution, F1, F2, F8, and F9.

	Hela (μM)	A549 (μM)	Particle Size (nm)	EE (%)
MPa	0.81	0.57	N/A	N/A
F1	1.00	0.65	105.83 ± 0.68	64.87 ± 2.93
F2	0.73	0.99	187.63 ± 1.02	22.35 ± 0.90
F8	1.95	2.21	221.10 ± 3.54	48.68 ± 0.04
F9	0.75	0.65	105.43 ± 0.92	25.91 ± 0.83

N/A, not applicable.

## Data Availability

The data presented in this study are available in [Design and Characterisation of pH-Responsive Photosensitiser-Loaded Nano-Transfersomes for Enhanced Photodynamic Therapy].

## Supplementary Materials

The following supporting information can be downloaded at: https://www.mdpi.com/article/10.3390/pharmaceutics14010210/s1, Table S1: Dark cytotoxicity against HeLa cell, Table S2: Light cytotoxicity against HeLa cell, Table S3: Dark cytotoxicity against A549 cell, Table S4: Light cytotoxicity against A549 cell.
