# Supplementary material for: Design and Characterisation of pH-Responsive Photosensitiser-Loaded Nano-Transfersomes for Enhanced Photodynamic Therapy
Source: Pharmaceutics. 2022 Jan 16;14(1):210. doi: 10.3390/pharmaceutics14010210 (PMC8781809; doi:10.3390/pharmaceutics14010210)
Supplement: Supplementary file 1 [file pharmaceutics-14-00210-s001.zip › pharmaceutics-1547648-supplementary.pdf]

pharmaceutics

Supplementary Materials: Design and Characterisation of pH-Responsive Photosensitiser-Loaded Nano-Transfersomes for Enhanced Photodynamic Therapy

Sooho Yeo, Il Yoon and Woo Kyoung Lee

Supplementary Information for In Vitro Photo-Irritation Studies

Table S1. Cell viability (%) of free MPa solution, F1, F2, F8, and F9 for dark cytotoxicity against HeLa cell. The concentration range of all compounds was 1.0 to 10.0 μM. The percentage of cell viability was determined by WST assay. Error values represent the standard deviation of three replicate experiments.

Concentration (μM)	1.0	2.5	5.0	10.0
MPa	116.1 ± 20.4	124.1 ± 21.6	106.8 ± 8.2	92.2 ± 6.0
F1	100.9 ± 5.3	95.9 ± 2.9	98.0 ± 7.1	92.3 ± 8.8
F2	106.9 ± 9.1	100.2 ± 2.1	83.4 ± 7.4	84.8 ± 13.5
F8	131.5 ± 5.0	128.7 ± 4.3	121.5 ± 1.3	84.9 ± 2.3
F9	143.0 ± 6.8	136.1 ± 11.5	130.0 ± 3.6	108.6 ± 12.9

Table S2. Cell viability (%) of free MPa solution, F1, F2, F8, and F9 for light cytotoxicity against HeLa cell. The concentration range of all compounds was 1.0 to 10.0 μM. The percentage of cell viability was determined by WST assay. Error values represent the standard deviation of three replicate experiments.

Concentration (μM)	1.0	2.5	5.0	10.0
MPa	38.2 ± 2.1	37.0 ± 1.0	38.8 ± 2.3	35.7 ± 1.3
F1	49.9 ± 4.8	27.6 ± 0.7	35.5 ± 2.8	33.7 ± 0.9
F2	31.9 ± 1.1	25.6 ± 6.4	35.4 ± 0.9	35.4 ± 1.2
F8	75.2 ± 2.7	35.2 ± 0.0	35.4 ± 1.3	36.1 ± 3.3
F9	33.3 ± 1.1	33.1 ± 0.4	34.0 ± 2.2	32.4 ± 2.1

Table S3. Cell viability (%) of free MPa solution, F1, F2, F8, and F9 for dark cytotoxicity against A549 cell. The concentration range of all compounds was 1.0 to 10.0 μM. The percentage of cell viability was determined by WST assay. Error values represent the standard deviation of three replicate experiments.

Concentration (μM)	1.0	2.5	5.0	10.0
MPa	98.6 ± 4.2	110.5 ± 10.2	96.0 ± 4.4	97.9 ± 9.7
F1	95.7 ± 3.0	91.3 ± 12.3	88.9 ± 3.1	86.6 ± 3.7
F2	90.6 ± 6.8	90.7 ± 7.1	98.1 ± 3.8	97.5 ± 0.6
F8	99.0 ± 5.6	97.7 ± 7.5	94.8 ± 1.9	87.4 ± 10.3
F9	108.2 ± 12.1	98.0 ± 4.1	91.0 ± 2.0	83.6 ± 5.9

Table S4. Cell viability (%) of free MPa solution, F1, F2, F8, and F9 for light cytotoxicity against A549 cell. The concentration range of all compounds was 1.0 to 10.0 μM. The percentage of cell viability was determined by WST assay. Error values represent the standard deviation of three replicate experiments.

Concentration (μM)	1.0	2.5	5.0	10.0
MPa	12.7 ± 1.0	15.2 ± 2.4	17.3 ± 1.3	15.6 ± 2.1
F1	23.3 ± 8.8	14.6 ± 1.8	15.0 ± 0.4	15.5 ± 0.2
F2	49.5 ± 5.2	23.9 ± 3.1	15.5 ± 1.8	15.5 ± 1.2
F8	106.5 ± 14.5	36.2 ± 5.9	19.0 ± 4.7	15.8 ± 0.7
F9	23.0 ± 6.0	18.2 ± 3.1	17.8 ± 2.1	21.6 ± 3.2
